# Physical activity and exercise for the prevention and management of mild cognitive impairment and dementia: a collaborative international guideline

**DOI:** 10.1007/s41999-023-00858-y

**Published:** 2023-09-28

**Authors:** Nicola Veronese, Pinar Soysal, Jacopo Demurtas, Marco Solmi, Olivier Bruyère, Nikos Christodoulou, Rodrigo Ramalho, Paolo Fusar-Poli, Andreas S. Lappas, Daniel Pinto, Kristian Steen Frederiksen, Grazia Maria Corbi, Olga Karpenko, Jean Georges, João Durães, Mathias Schlögl, Ozlem Yilmaz, Cornel Sieber, Susan D. Shenkin, Lee Smith, Jean-Yves Reginster, Stefania Maggi, Federica Limongi, Joan Ars, Mario Barbagallo, Antonio Cherubini, Terry Quinn, on behalf of Alzheimer Europe, Jean Georges, European College of Neuropsychopharmacology, Paolo Fusar-Poli, Marco Solmi, European Geriatric Medicine Society (Lead Society), Javier Alonso Ramirez, Mariana Alves, Gülistan Bahat, Jurgen Bauer, Ivan Bautman, Rui Buzaco, Álvaro Casas Herrero, Matteo Cesari, Yaohua Chen, Antonio Cherubini, Grazia Maria Corbi, Alfonso Cruz Jentoft, Anne-Marie De Cock, Jacopo Demurtas, Burcu Balam Dogu, Ellen Freiberger, Miriam L. Haaksma, Marina Kotsani, Sarah Lamb, Mounir Lamloum, Evelyne Liuu, Rene Melis, Laura Monica Perez Bazan, Maria Cristina Polidori, Joan Ars Ricart, Almudena Medina Rincon, Yves Rolland, Roman Romero-Ortuno, Guillaume Sacco, Mathias Schlögl, Daniel Schoene, Debbie Shapiro, Susan Shenkin, Cornel Sieber, Lee Smith, Pinar Soysal, Thomas Tannou, Nicola Veronese, Ozlem Yilmaz, European Interdisciplinary Council on Ageing, Agar Brugiavini, Federica Limongi, Stefania Maggi, European Society of Clinical and Economic Aspects of Osteoporosis and Osteoarthritis, Olivier Bruyère, Daniel Pinto, Jean-Yves Reginster, International Association of Gerontology and Geriatrics-European Region, Mario Barbagallo, Scottish Brain Health ARC, World Psychiatry Association-Preventive Psychiatry Section, Nikos Christodoulou, Olga Karpenko, Andreas S. Lappas, Rodrigo Ramalho, endorsed by the European Academy of Neurology, Joao Duraes, Reinhold Schmidt, Kristian Steen Frederiksen

**Affiliations:** 1 Geriatric Unit, Department of Internal Medicine and Geriatrics, University of Palermo, Via del Vespro, 141, 90127 Palermo, Italy; 2 Faculty of Medicine, Department of Geriatric Medicine, Bezmialem Vakif University, Istanbul, Turkey; 3 Clinical and Experimental Medicine PhD Program, Università di Modena e Reggio Emilia, Modena - Azienda USL Sud Est Toscana, Grosseto, Italy; 4 Department of Psychiatry, University of Ottawa, Ottawa, ON Canada; 5 Department of Mental Health, The Ottawa Hospital, Ottawa, ON Canada; 6 Department of Child and Adolescent Psychiatry, Charité Universitätsmedizin, Berlin, Germany; 7 Division of Public Health, Epidemiology and Health Economics, World Health Organization, Collaborating Center for Epidemiology of Musculoskeletal Health and Aging, University of Liège, Liège, Belgium; 8 Department of Psychiatry, University of Thessaly Medical School, Volos, Greece; 9 World Psychiatric Association, Section of Preventive Psychiatry, University of Nottingham Medical School, Nottingham, UK; 10 Department of Social and Community Health, School of Population Health, The University of Auckland, Auckland, New Zealand; 11 Department of Psychosis Studies, King’s College London, London, UK; 12 Department of Brain and Behavioral Sciences, University of Pavia, Pavia, Italy; 13 Faculty of Medicine, Department of Psychiatry, University of Thessaly, Larissa, Greece; 14 Aneurin Bevan University Health Board, Newport, Wales, UK; 15 Department of Physical Therapy, College of Health Sciences, Marquette University, Milwaukee, WI USA; 16 Department of Neurology, Danish Dementia Research Centre, Copenhagen University Hospital Rigshospitalet, Copenhagen, Denmark; 17 Department of Translational Medical Sciences, University of Naples “Federico II”, Naples, Italy; 18 Chair of the WPA Preventive Psychiatry Section, Mental-Health Clinic No. 1 Named After N.A. Alexeev, Moscow, Russia; 19 Alzheimer Europe, Luxembourg, Luxembourg; 20 Neurology Department, Coimbra University Hospital Centre, Coimbra, Portugal; 21 Faculty of Medicine, Coimbra University, Coimbra, Portugal; 22 Division of Geriatric Medicine, Clinic Barmelweid, Barmelweid, Switzerland; 23 Department of Geriatric Medicine, Istanbul Training and Research Hospital, Samatya, Istanbul, Turkey; 24 Institute for Biomedicine of Aging, Friedrich-Alexander-Universität Erlangen-Nürnberg, Kobergerstr. 60, 90408 Nuremberg, Germany; 25 Department of Medicine, Kantonsspital Winterthur, Brauerstrasse 15, Postfach 834, 8401 Winterthur, Zurich Switzerland; 26 Ageing and Health Research Group and Advanced Care Research Centre, Usher Institute, University of Edinburgh, Edinburgh, Scotland, UK; 27 Centre for Health Performance and Wellbeing, Anglia Ruskin University, Cambridge, UK; 28 Division of Public Health, Epidemiology and Health Economics, University of Liège, Liège, Belgium; 29 National Research Council, Neuroscience Institute, Aging Branch, Padua, Italy; 30 RE-FiT Barcelona Research Group, Vall d’Hebron Institute of Research (VHIR) and Parc Sanitari Pere Virgili, Barcelona, Spain; 31 Aging Research Center, Department of Neurobiology, Care Sciences and Society (NVS), Karolinska Institutet and Stockholm University, Stockholm, Sweden; 32 Geriatria, Accettazione Geriatrica e Centro di Ricerca Per l’invecchiamento, IRCCS INRCA, Ancona, Italy; 33 Department of Geriatric Medicine, Glasgow Royal Infirmary, Glasgow, UK; 34 Institute of Cardiovascular and Medical Sciences, University of Glasgow, Glasgow, UK

**Keywords:** Cognition, Dementia, Mild cognitive impairment, Guidelines, Physical activity, Exercise, Older adult

## Abstract

**Background:**

Physical activity and exercise have been suggested as effective interventions for the prevention and management of mild cognitive impairment (MCI) and dementia, but there are no international guidelines.

**Objectives:**

To create a set of evidence- and expert consensus-based prevention and management recommendations regarding physical activity (any bodily movement produced by skeletal muscles that results in energy expenditure) and exercise (a subset of physical activity that is planned, structured, repetitive), applicable to a range of individuals from healthy older adults to those with MCI/dementia.

**Methods:**

Guideline content was developed with input from several scientific and lay representatives’ societies. A systematic search across multidisciplinary databases was carried out until October 2021. Recommendations for prevention and management were developed according to the GRADE and complemented by consensus statements from the expert panels.

**Recommendations:**

Physical activity may be considered for the primary prevention of dementia. In people with MCI there is continued uncertainty about the role of physical activity in slowing the conversion to dementia. Mind–body interventions have the greatest supporting evidence. In people with moderate dementia, exercise may be used for maintaining disability and cognition. All these recommendations were based on a very low/low certainty of evidence.

**Conclusions:**

Although the scientific evidence on the beneficial role of physical activity and exercise in preserving cognitive functions in subjects with normal cognition, MCI or dementia is inconclusive, this panel, composed of scientific societies and other stakeholders, recommends their implementation based on their beneficial effects on almost all facets of health.

**Supplementary Information:**

The online version contains supplementary material available at 10.1007/s41999-023-00858-y.

## Introduction

The number of people with dementia worldwide was estimated at 47.5 million in 2015, and is likely to reach 75.6 million by 2030 [1]. Future projections indicate that this number will increase to 135.46 million in 2050, [1] with approximately 7.7 million new cases of dementia each year [1]. People with mild cognitive impairment (MCI) are at greater risk of dementia than the general population, and the annual progression rate ranges from 10% to 15% [2, 3].

Unfortunately, there are no definitive disease modifying treatments for dementia, so epidemiological research may highlight modifiable targets for prevention [4]. Physical activity is one promising target [5]. It has been estimated that 3% of dementia cases could be prevented by increasing levels of free-living physical activity [6–8], and a growing body of literature reports the importance of physical activity (i.e., any bodily movement produced by skeletal muscles that results in energy expenditure) and exercise (i.e., a subset of physical activity that is planned, structured, and repetitive) for preventing and slowing down the pathological processes and dementia-related problems [9]. In this regard, older people who are physically active are more likely to maintain cognition than those who are not [6]. The important role of physical activity has also been highlighted for people already living with dementia. Indeed, exercise helps improving important outcomes, such as cognition [10]. Moreover, physical activity in general and exercise interventions in particular might help ameliorate Behavioural and Psychological Symptoms in Dementia (BPSD) [11].

While these data are encouraging, not all studies have shown an independent association. Similarly, the strength of the association is unclear, and a causal relationship between physical activity and cognitive outcomes is still debated. Where randomised clinical trial evidence is available, results are not consistent. For example, a recent large trial of people with dementia has reported worsening in some aspects of cognition after moderate-to-high intensity multicomponent exercise [12]. The effect of physical activity/exercise on MCI is also unclear. Some studies reported that physical activity/exercise could prevent the transition from MCI to dementia, and that these interventions can improve cognitive and non-cognitive outcomes in this population [13].

Specific guidelines regarding physical activity/exercise for preventing or managing dementia and MCI are currently not available. Moreover, although observational evidence generally supports an association between physical activity/exercise and cognitive outcomes, intervention studies are less common and definitive results are still not present. Finally, the lack of recommendations on exercise and physical activity in dementia guidelines is another relevant limitation. Given this background, we aimed to develop specific guidelines by combining published literature and expert consensus regarding this topic, and involving international, mainly European, scientific and lay representatives’societies [14].

## Methods

The protocol was published on 01st May 2022 at https://www.eugms.org/fileadmin/user_upload/Special_Interest_Group_Documents/Protocol_for_guidelines_phys_activity_dementia.pdf and it is freely available.

### Guideline development group

The names and surnames of the participants, including their role, are presented in Supplementary Table 1.

Briefly:

- A Committee was formed comprising the Presidents of each society (or a representative).
- The Chairperson for the Guidelines named by the European Geriatric Medicine Society (Veronese) revised the questions proposed.
- The Committee appointed five leaders (Solmi, Bruyère, Soysal, Pinto, Frederiksen) as chairs of each of the topics of the guidelines, i.e., the role of physical activity/exercise in primary prevention, MCI, and dementia, respectively.
- The work was divided in three groups composed by the leaders, at least one representative of each society involved and one lay representative.
- The leaders, the chairperson of the guidelines, and one expert of each society not previously involved in the manuscript drafting discussed the recommendations during an online meeting (01st April 2023) and agreement was reached through discussion. The votes of the members were expressed anonymously to allow for free expression of views using an online form. Consensus on each question/intervention was defined if at least 80% of the members of the working group were either “strongly” or “weakly” in favour or against a recommendation [15].

### Development of the questions for guideline: PICO

The PICO (Participants, Intervention, Control, Outcomes) questions are fully reported, by each topic (prevention, MCI, dementia) in Supplementary Table 2. Briefly, the leaders of the topics, together with the Chairperson of the Guidelines and an expert in methodology (Quinn) produced relevant PICO questions (within the three areas of interest), which were subsequently circulated amongst leaders and presidents/representatives of the societies. The associations of the lay representatives were actively involved and revised the three PICO questions. These three working groups met online separately.

Regarding the participants, we categorised three populations: those not initially affected by MCI or dementia, ‘MCI’, and ‘dementia’. For populations not affected by MCI or dementia, we accepted any paper with the intention of including only people with no established cognitive diagnosis and where the study took reasonable steps to ensure that the population was free of people living with a cognitive syndrome. We accepted any validated method of diagnosis for dementia and MCI, these could include medical records, cognitive testing against pre-defined standards, and clinical diagnosis using DSM criteria or similar. We included all cause cognitive syndromes as well as diagnoses of specific diseases such as Alzheimer’s disease (AD) and noted this as part of the data extraction.

Regarding the intervention, a technical online meeting was held for defining physical activity and exercise with experts from the EuGMS and other societies. The experts indicated that all types of physical activity and exercise must be included in these guidelines. Physical activity was defined as any bodily movement produced by skeletal muscles that results in energy expenditure [16], while exercise was a subset of physical activity that is planned, structured, and repetitive and has as a final or an intermediate objective for the improvement or maintenance of physical fitness [16]. These definitions were made in agreement with those indicated by the World Health Organization [16]. Physical activity was classified as in high, moderate, low according to the original definition reported in the work; exercise in aerobic, anaerobic, mixed further detailing in running, cycling, swimming, brisk walking, dancing, walking, push-ups, pull-ups, lunges, squats, bench press, weight training, functional training, eccentric training, interval training, sprinting, high-intensity interval training, based on their nature.

The working groups recommended selecting inactive subjects (usual care, standard care, or waiting list) as controls in intervention studies and individuals with lower physical activity levels from the lowest quantile available in observational studies. Consequently, studies that included active control groups (e.g., nutritional interventions) were excluded.

The list of the most relevant outcomes was proposed by the three working groups separately, based on the literature and their clinical experience. The outcomes were then divided into primary or secondary, based on their importance, as fully reported in the Supplementary Table 2. Working groups scored a list of potential outcomes and only those where there was consensus on importance were included as ‘primary’.

Finally, regarding the study design, we used a hierarchical approach favouring systematic reviews (with or without meta-analyses) that synthesized randomized controlled trials (RCTs) or controlled clinical trials (CCTs) as priority. If not available, singular RCTs/CCTs were used. Systematic reviews without meta-analysis were reported as narrative findings. In the case of no systematic review available for one of the review questions, or only systematic reviews over 3 years, we ran additional searches for primary studies and subsequently integrated the primary findings.

### Search strategy

The literature searching was carried out by two expert librarians according to the guidance prescribed by The Cochrane Handbook [17] using several databases (MEDLINE, Embase, The Cochrane Library, Epistemonikos) interrogated using Ovid, from databases’ inception to 09th October 2021. Supplementary Table 3 reports the search strategy proposed in Medline. The search was then adapted to the other databases.

### Study selection

Studies identified were screened by two people, independently, using COVIDENCE (https://www.covidence.org/) in a two-step approach, with an initial screening carried out on title and abstract level followed by a second step in which full texts of the studies identified were screened. Any conflicts were resolved by one of the two leaders of the group. When more than one systematic review/meta-analysis assessed the same outcome in the same population, we only included the one with the largest number of studies.

### Data extraction

Data from included studies were extracted by one member of each of the three teams, checked by another independent member, in a piloted Microsoft Excel spreadsheet. The Excel spreadsheet was initially piloted in double blinded fashion, using one eligible meta-analysis.

Data extraction was made using a two-step approach. First, at the level of the systematic review/meta-analysis, reported effect sizes and number of studies included were extracted; second, at single study level, considering the results of the studies evaluated in the systematic reviews and meta-analyses. For each systematic review and meta-analysis, we extracted: the number of studies, the number of participants in each arm, participant demographics, the length of follow-up, details of physical activity/exercise intervention (in terms of type, frequency, intensity, motivation, person responsible for delivering the intervention), effect size of outcomes of interest. Data regarding the data quality assessment was also extracted.

### Risk of bias

Two reviewers assessed the risk of bias of the included systematic reviews and meta-analyses using ROBIS (Risk of Bias Assessment Tool for Systematic Reviews) [18]. ROBIS includes four different domains: domain 1, study eligibility criteria; domain 2, identification and selection of studies; domain 3, data collection and study appraisal; domain 4, synthesis and findings. For single RCTs and CCTs, we used the Cochrane risk-of-bias tool for randomized trials (RoB) [17]; the Newcastle Ottawa Scale (NOS) [19] was used for assessing quality of observational studies. The ROBIS for eligible systematic reviews and meta-analyses is graphically reported in Supplementary Fig. 1. Since several systematic reviews included data for both MCI and dementia, they were evaluated together.

### Data synthesis and evaluation of the evidence

For each meta-analysis, we estimated the summary effect size and its 95% confidence interval (CI) by using a random-effects, with the DerSirmonian–Leird’s method [20]. Between-study inconsistency was estimated with the *I*^*2*^ metric, with values ≥ 50% indicative of high heterogeneity [21]. All statistical analyses were conducted in Stata, version 14.0 (StataCorp).

### Evaluation of the quality of evidence and formulation of recommendations

Evidence from meta-analyses was evaluated using the GRADE (Grading of Recommendations, Assessment, Development and Evaluation) assessment. The GRADE framework takes into account several important domains in the certainty of the evidence, including study design, risk of bias, inconsistency, indirectness, imprecision and other aspects, such as publication bias [22].

The GRADE assessment was carried out by three investigators (Demurtas, Veronese, Pinto) and checked and corrected, if needed, by two others (Solmi, Quinn). Supplementary Table 4 reports the criteria used, for each domain, for performing the GRADE. The certainty of the evidence was then reported as: very low (the true effect is probably markedly different from the estimated effect), low (the true effect might be markedly different from the estimated effect), moderate (the true effect is probably close to the estimated effect) or high (there is a lot of confidence that the true effect is similar to the estimated effect) [22]. The results of data analysis were imported into the GRADEpro Guideline Development Tool (McMaster University, 2015; developed by Evidence Prime, Inc.) “Evidence-based Recommendations” were based on the GRADE methodology. The direction, strength and formulation of the recommendations were determined according to the GRADE evidence profiles. The quality of the evidence was graded according to the GRADE from very low to high; the strength of the recommendation was based on the literature and the experts’ opinion supporting the sentence graded from strong to weak [23].

Finally, Expert Consensus Statements were added whenever the PICO group considered that there was insufficient evidence available to provide Evidence-based Recommendations and where practical guidance is needed for routine clinical practice. The Expert Consensus Statements were based on voting by all expert members.

### Target population

Stakeholders, in terms of lay representatives, were involved in the guideline development. The guidelines were developed for use by all health and social care professionals (medical and non-medical) dealing with dementia and MCI in their clinical practice, including specialists, family physicians, clinical or institutional leaders/administrators, as well as patients and their caregivers. These guidelines aim to inform clinical decisions, policy, and standards of care, particularly in terms of a public health perspective.

### Internal and external review

The drafts were all cross checked by methodology and topic experts from within the group who had not been involved in the primary analyses in an internal review step, during two rounds. The external review was guaranteed by the Reviewers of the European Geriatric Medicine and by the experts of the European Academy of Neurology, not involved in the preparation of the manuscript.

## Results

### Topic one: prevention

#### PICO question: In people without dementia or MCI, are physical activity and/or exercise able to delay the onset of dementia and/or MCI?

##### Analysis of current evidence

In this PICO question, the important aspect of physical activity/exercise as preventive measure for dementia or MCI was addressed. To prevent dementia in people still not affected by this condition is a public health priority. An experts’ consensus, for example, has suggested that second-generation memory clinics (Brain Health Services) should focus not only in the management of people with dementia, but also in evidence-based and ethical dementia prevention in at-risk individuals [24]. In this context, physical activity and a structured exercise program were highly encouraged for the prevention of dementia [24].

In these guidelines, for the topic of prevention, the incidence of dementia (any or specific) was considered the primary outcome. We found a large systematic review, of good quality according to ROBIS, with a meta-analysis of 49 observational cohort studies and a total of 257,983 participants free from dementia or MCI at baseline [25]. This work overall reported [25] that higher self-reported physical activity levels were associated with a significantly lower risk of any dementia, AD (Alzheimer’s disease), or vascular dementia, with a dose-gradient response. However, the low quality of the studies included, the high heterogeneity, and the presence of publication bias must be acknowledged as important limitations [25].

Regarding exercise, we found only one large RCT dealing with the outcomes of interest, with a low risk of bias according to the Cochrane RoB tool [26]. In the context of the Lifestyle Interventions and Independence for Elders (LIFE) study that enrolled 1635 community-living participants without evidence of cognitive disorders at baseline, over 24 months of follow-up, a moderate intensity physical activity program compared to a health education program did not result in a lower incidence of MCI or dementia [26]. This result was somewhat expected, because, as mentioned by the same authors, incidence of MCI and dementia were only tertiary outcomes, and therefore, this study was probably underpowered for investigating these specific endpoints. While the trial did not suggest a beneficial effect of exercise over health education, there was also no suggestion of any harm.

##### Recommendations

In people without any evidence of dementia or MCI, physical activity may be considered for the primary prevention of dementia, AD, or vascular dementia.

In participants without dementia or MCI, exercise may be no better than health education for the primary prevention of dementia and MCI.

*Quality of evidence*: Very low ⊕ for physical activity; very low ⊕ for exercise.

*Strength of recommendation*: Strong for intervention ↑↑ for physical activity; strong for intervention ↑↑ for exercise.

##### Additional information/secondary outcomes

No studies met the eligibility criteria for secondary outcomes, i.e., adverse events (total and specific) and safety measures, drop-out rate, disability in ADL (activities of daily living)/IADL (instrumental activities of daily living): global and specific domains of cognition (i.e., attention, executive function, memory, motor speed, and language), or quality of life, not included as primary outcomes.

##### Expert consensus statement

100% of the experts agreed that physical activity may delay the onset of dementia (any and specific cause) but evidence is uncertain, and physical activity should be considered as part of a multicomponent intervention (Table 1). 100% agreed that exercise alone may be no better than health education for the primary prevention of dementia and MCI. However, the two interventions may be complementary (Table 2).

**Table 1:** Effect of high physical activity levels on incident mild cognitive impairment and dementia

Certainty assessment							No. of patients	Effect		Certainty	Importance
No. of studies	Study design	Risk of bias	Inconsistency	Indirectness	Imprecision	Other considerations		Relative (95% CI)	Absolute (95% CI)		
Any dementia											
49	Observational studies	Very serious^a^	Serious^b^	Not serious	Not serious	Publication bias strongly suspected^c^; dose response gradient	257,983	RR 0.80 (0.77–0.84)	–	⨁◯◯◯ Very low	Critical
Alzheimer’s disease											
24	Observational studies	Very serious^a^	Not serious	Not serious	Not serious	Publication bias strongly suspected^c^; dose response gradient	128,261	RR 0.86 (0.80–0.93)	**–**	⨁◯◯◯ Very low	Critical
Vascular dementia											
24	Observational studies	Very serious^a^	Not serious	Not serious	Not serious	Publication bias strongly suspected^c^; dose response gradient	33,870	RR 0.79 (0.66–0.95)	**–**	⨁◯◯◯ Very low	Critical

*CI* confidence interval, *RR* risk ratio

^a^Risk of bias present in more than 30% of the studies included

^b^I2 between 50% and 75%

^c^Publication bias reported

**Table 2:** Effect of exercise on incident mild cognitive impairment and dementia

Certainty assessment							No. of patients		Effect		Certainty	Importance
No. of studies	Study design	Risk of bias	Inconsistency	Indirectness	Imprecision	Other considerations	Intervention	Controls	Relative (95% CI)	Absolute (95% CI)		
Any dementia												
1	Randomized controlled trial	Not serious	Not serious	None	Very serious^a^	None	28/818 (3.4%)	29/817 (3.5%)	OR 0.96 (0.57–1.63)	1 fewer per 1.000 (from 15 fewer to 21 more)	⨁◯◯◯ Very low	Critical
Mild cognitive impairment												
1	Randomized controlled trial	Not serious	Not serious	None	Very serious^a^	None	70/686 (10.2%)	62/682 (9.1%)	OR 1.14 (0.79–1.62)	11 more per 1.000 (from 18 fewer to 49 more)	⨁◯◯◯ Very low	Critical

*CI* confidence interval, *OR* odds ratio

^a^Only one study with wide confidence intervals

##### Future research directions

- There is a need for adequately powered RCTs evaluating the effect of exercise and physical activity for the primary prevention of MCI and dementia and for improving cognitive outcomes.
- Studies using multicomponent comprehensive interventions are urgently needed for exploring the role of physical activity and exercise in the context of other comprehensive approaches for the primary prevention of dementia and MCI.
- The implementation of physical activity and exercise in people free from dementia and MCI is of importance also from a public health perspective, including economic aspects.

### Topic two: mild cognitive impairment (MCI)

#### PICO question: Are physical activity and exercise able to delay the onset of dementia in people with MCI?

##### Analysis of current evidence

MCI may be an early window for treatment for preventing or delaying dementia onset [27] . Conflicting epidemiological evidence supports the idea that MCI could be considered a potential risk factor for dementia, since it is estimated that the rate of conversion to dementia in the MCI population is equal to 10–15% per year [28] compared to 1–2% in people without MCI [29]. Physical inactivity seems an independent risk factor for the conversion from MCI to dementia [30], even if its role is still largely debated. Some authors proposed a positive effect of exercise for delaying the onset of dementia in people with MCI [31, 32].

Supplementary Table 2 indicates all the outcomes considered for the PICO questions. In these guidelines, we were not able to find any high-quality systematic reviews of trials or single RCT or non-randomized evidence able to indicate that physical activity or exercise can delay the onset of dementia in people with MCI that was considered our primary outcome. We found a single observational study that followed 247,149 individuals with MCI in Korea [33]. Compared to people who never reported physical activity, ‘maintenance’ of physical activity throughout 6 years surrounding MCI diagnosis was associated with a significantly lower risk of conversion from MCI to dementia [33]. Likewise, those who initiated physical activity after MCI diagnosis also had a significantly lower risk of conversion versus those who never engaged in physical activity [33].

Regarding secondary outcomes, we found an umbrella review on the topic of exercise in MCI for improving cognitive outcomes, including RCTs [34], adding the GRADE evaluation also for non-statistically significantly outcomes. The umbrella review contains five systematic reviews with meta-analysis regarding the impact of physical activity/exercise in MCI on cognitive outcomes [35–39]. Of these, five meta-analyses only one [36] was rated as low risk of bias, according to the ROBIS, while the other fours at high risk of bias. The limited information regarding study eligibility and identification of the studies was the main reason of the high risk of bias of the meta-analyses of this topic. The team was not able to find any new study that could contribute additional findings to the meta-analyses conducted more than 3 years ago.

Overall, exercise interventions were highly heterogeneous in terms of type, frequency, duration, and intensity across the studies included. Regarding mind–body interventions (i.e., a kind of intervention that includes a mental health perspective, such as Tai Chi and yoga) [40] (mean frequency: 3 times/weekly; session: 30–90 min, each one; mainly in group), we observed a small effect on global cognition (SMD = 0.36; 95% CI 0.20–0.52; low certainty), short-term memory (SMD = 0.74; 95% CI 0.57–0.91; low certainty), executive function (SMD = – 0.42; 95% CI 0.63–0.21; low certainty), visuospatial executive function (SMD = 0.36; 95% CI 0.07–0.64; low certainty), and attention (SMD = 0.39; 95% CI 0.07–0.72; low certainty). In particular, Tai Chi was able to maintain stable short memory compared to control group (SMD = 0.77; 95% CI 0.45–1.09; very low certainty). Resistance training (mean frequency: 2 times/weekly; mean session duration: 60 min) had a large effect on global cognition (SMD = 0.80; 95% CI 0.29–1.31; very low certainty).

Moreover, it seems that another type of exercise, i.e. mixed aerobic and anaerobic exercise, was able to affect global cognition (SMD = 0.30; 95% CI 0.11–0.49; moderate certainty), but not able to modify some specific cognitive domains, such as attention, measured with the Stroop test, immediate recall, working or delayed memory (high certainty of evidence according to the GRADE for all these domains). Similarly, aerobic exercise did not substantially affect immediate recall (moderate certainty of evidence according to the GRADE), executive function (moderate certainty of evidence), attention (high certainty of evidence), and verbal fluency (high certainty of evidence), although it maintained stable delayed memory (SMD = 0.26; 95% CI 0.06–0.46; moderate certainty of evidence).

##### Recommendations

In people with MCI there is continued uncertainty about the role of physical activity and exercise in slowing the conversion to dementia.

*Quality of evidence*: Very low ⊕ for physical activity; very low ⊕ for exercise.

*Strength of recommendation*: Strong for intervention ↑↑ for physical activity; strong for intervention ↑↑ for exercise.

##### Additional information

In people with MCI, mixed physical activity/exercise did not significantly change IADL scores compared to standard care (high risk of bias according to the ROBIS) [41], this effect was largely expected, since the functional aspect is one of the essential points for differentiating people with MCI and people with dementia [42]. The studies included in our guidelines did not report any information regarding quality of life or side effects.

##### Expert consensus statement

100% of the experts agreed that MCI should not discourage exercise (Table 3).

**Table 3:** Effect of physical activity on dementia incidence in people with mild cognitive impairment at baseline

Certainty assessment							No. of patients	Effect	Certainty	Importance
No. of studies	Study design	Risk of bias	Inconsistency	Indirectness	Imprecision	Other considerations		Relative (95% CI)		
Conversion to dementia										
1	Observational study	Very serious^a^	Not serious	Not serious	Serious^b^	None	247,149	Reference “never PA” Adj HR 0.89 (0.85–0.93) (initiation-PA) Adj HR 1.00 (0.96–1.04) (Withdrawal-PA) Adj HR 0.82 (0.79–0.86) (Maintenance-PA)	⨁◯◯◯ Very low	Critical

^a^One study at high risk of bias

^b^Only one study

There is no form of exercise that seems to be superior for preventing or delaying cognitive decline in people living with MCI (Table 4).

**Table 4:** Effect of exercise on cognitive outcomes in mild cognitive impairment

Certainty assessment							No. of patients		Effect		Certainty	Importance
No. of studies	Study design	Risk of bias	Inconsistency	Indirectness	Imprecision	Other considerations	Intervention	Standard care	Relative (95% CI)	Absolute (95% CI)		
Short-term memory (mind body intervention)												
12	Randomized controlled trials	Very serious^a^	Not serious	Not serious	Not serious	None	356	387	–	SMD 0.74 SD higher (0.57 higher to 0.91 higher)	⨁⨁◯◯ Low	Important
Short-term memory (Tai Chi intervention)												
4	Randomized controlled trials	Very serious^a^	Not serious	Not serious	Serious^d^	None	114	112	–	SMD 0.77 SD higher (0.45 higher to 1.09 higher)	⨁◯◯◯ Very low	Important
Global cognition (mind body intervention)												
9	Randomized controlled trials	Very serious^a^	Not serious	Not serious	Not serious	None	425	557	–	SMD 0.36 SD higher (0.2 higher to 0.52 higher)	⨁⨁◯◯ Low	Important
Executive function (mind body intervention)												
9	Randomized controlled trials	Very serious^a^	Not serious	Not serious	Not serious	None	426	474	–	SMD 0.42 SD lower (0.63 lower to 0.21 lower)	⨁⨁◯◯ Low	Important
Global cognition (mixed physical activity intervention)												
8	Randomized controlled trials	Serious^e^	Not serious	Not serious	Not serious	None	347	316	–	SMD 0.3 SD higher (0.11 higher to 0.49 higher)	⨁⨁⨁◯ Moderate	Important
Global cognition (resistance training intervention)												
4	Randomized controlled trials	Very serious^a^	Serious^f^	Not serious	Serious^d^	None	77	69	–	SMD 0.8 SD higher (0.29 higher to 1.31 higher)	⨁◯◯◯ Very low	Important
Visuospatial executive function (mind body intervention)												
4	Randomized controlled trials	Very serious^a^	Not serious	Not serious	Not serious	None	163	162	–	SMD 0.36 SD higher (0.07 higher to 0.64 higher)	⨁⨁◯◯ Low	Important
Delayed memory (aerobic exercise intervention)												
7	Randomized controlled trials	Not serious	Serious^f^	Not serious	Not serious	None	638	675	–	SMD 0.26 SD higher (0.06 higher to 0.46 higher)	⨁⨁⨁◯ Moderate	Important
Attention (mind body intervention)												
5	Randomized controlled trials	Very serious^a^	Not serious	Not serious	Not serious	None	185	180	–	SMD 0.39 SD higher (0.07 higher to 0.72 higher)	⨁⨁◯◯ Low	Important
Processing speed (mind body intervention)												
4	Randomized controlled trials	Very serious^g,h^	Not serious	Not serious	Serious^d^	None	184	184	–	SMD 0.1 SD higher (0.005 lower to 0.63 higher)	⨁◯◯◯ Very low	Important
Immediate recall (aerobic exercise)												
6	Randomized controlled trials	Not serious	Serious^f^	Not serious	Not serious	None	338	339	–	SMD 0.26 SD higher (0.004 lower to 0.52 higher)	⨁⨁⨁◯ Moderate	Important
Attention (measured with TMT-B) (mixed physical activity intervention)												
7	Randomized controlled trials	Very serious^g^	Not serious	Not serious	Not serious	None	394	431	–	MD 6.77 (1.14 lower to 14.67 higher)	⨁⨁◯◯ Low	Important
Attention (measured with Stroop test) (mixed physical activity intervention)												
6	Randomized controlled trials	Not serious	Not serious	Not serious	Not serious	None	271	271	–	SMD 0.19 SD higher (0.03 lower to 0.4 higher)	⨁⨁⨁⨁ High	Important
Immediate recall (mixed physical activity intervention)												
9	Randomized controlled trials	Not serious	Not serious	Not serious	Not serious	None	396	395	–	0.11 higher (0.07 lower to 0.27 higher)	⨁⨁⨁⨁ High	Important
Verbal fluency (mixed physical activity intervention)												
8	Randomized controlled trials	Not serious	Serious^f^	Not serious	Not serious	Publication bias strongly suspected^h^	477	476	–	SMD 0.12 SD higher (0.14 lower to 0.38 higher)	⨁⨁◯◯ Low	Important
Working memory (mixed physical activity intervention)												
7	Randomized controlled trials	Not serious	Not serious	Not serious	Not serious	None	361	331	–	SMD 0.57 SD higher (1.21 lower to 2.34 higher)	⨁⨁⨁⨁ High	Important
Executive function (aerobic exercise)												
4	Randomized controlled trials	Not serious	Serious^f^	Not serious	Not serious	None	317	317	–	SMD 0.09 SD lower (0.38 lower to 0.2 higher)	⨁⨁⨁◯ Moderate	Important
Attention (aerobic exercise)												
4	Randomized controlled trials	Not serious	Not serious	Not serious	Not serious	None	375	374	–	SMD 0.06 SD higher (0.72 lower to 0.3 higher)	⨁⨁⨁⨁ High	Important
Verbal fluency (aerobic exercise)												
5	Randomized controlled trials	Not serious	Not serious	Not serious	Not serious	None	563	597	–	MD 0.16 lower (1.74 lower to 1.42 higher)	⨁⨁⨁⨁ High	Important
Delayed memory (mixed physical activity intervention)												
10	Randomized controlled trials	Not serious	Not serious	Not serious	Not serious	None	534	535	–	SMD 0.002 SD higher (0.14 lower to 0.14 higher)	⨁⨁⨁⨁ High	Important

*CI* Confidence interval, *STM* Short-Term Memory, SMD Standardized mean difference, *SD* Standard deviation, *TMTB* Trail Making Test B

^a^One or more of the three criteria (randomization, masking, drop-out rate > 30%) is not met in > 30% of trials included

^b^*I*^2^ ≥ 75%

^c^Egger's test (*p* value) < 0.0001

^d^Total sample size < 400 participants

^e^One or more of the three criteria (randomization, masking, drop-out rate < 30%) is not met in 10–30% of trials included

^f^*I*^2^ between 50% and 75%

Choice of exercise should be based on factors, such as comorbidity, and the preference of the person with MCI (Table 5).

**Table 5:** Effect of exercise on secondary outcomes in mild cognitive impairment

Intervention	Population	Outcome	Number of studies	Main findings
Mixed	MCI	Disability	3	In none of the studies, MCT was superior to active comparison or control interventions on IADL performance

*MCI* Mild Cognitive Impairment, *MCT* Multicomponent interventions, *IADL* Instrumental Activities of Daily Living

##### Future research directions

- There is a need for adequately powered randomized controlled trials evaluating the effect of exercise in people with MCI for the prevention of the onset of dementia, considered as primary outcome.
- Studies using multicomponent interventions are needed for exploring the role of physical activity and exercise in the context of other non-pharmacological approaches in people with MCI.
- Studies regarding the effect of physical activity and exercise on non-cognitive outcomes in people with MCI are needed.
- Further studies regarding aerobic and anaerobic exercise are needed, since the literature regarding these interventions and cognitive outcomes in MCI is conflicting.

### Topic three: dementia

#### PICO question: Is physical activity/exercise able to improve cognition and disability in people with dementia?

##### Analysis of current evidence

We found an umbrella review regarding the topic of exercise in dementia for improving cognitive and non-cognitive outcomes, including RCTs [34], adding the GRADE evaluation also for non-statistically significantly outcomes. The umbrella review contains ten systematic reviews with meta-analysis regarding the impact of physical activity/exercise in dementia [37, 43–51]. Only two meta-analyses [45, 49] had a low risk of bias, according to the ROBIS evaluation. Similarly, to the meta-analyses of MCI, limited information regarding study eligibility criteria and identification and selection of the studies were the main issues. The team was not able to find any new RCT able to add new findings in the meta-analyses older than 3 years.

Overall, in people with dementia, mixed physical activity/exercise (mean frequency: 2 times/weekly; mean session duration: 40 min) was effective in improving global cognition in moderate AD (mean Mini-Mental State Examination [MMSE] = 15.6, range 12–24) (SMD = 1.10; 95% CI 0.65–1.64; very low certainty according to the GRADE). A similar effect was observed in any dementia (mean MMSE of 15.6; range 5.8–24; mean frequency of the exercise: 2 times/weekly; mean session duration: 140 min) using global cognition as outcome (SMD = 0.48; 95% CI 0.22–0.74; low certainty). No effect of physical activity/exercise on specific cognitive domains such as attention, executive function, memory, motor speed, and language were observed in systematic reviews without meta-analysis. Moreover, home-based physical activity interventions in people with a moderate degree of dementia (mean MMSE = 18, range 14–22; mean frequency of the exercise: 3 times/weekly; mean session duration: 40 min) stabilized disability in activities of daily living (SMD = 0.77; 95% CI 0.17–1.37; low certainty of evidence).

##### Recommendation

In people with moderate dementia, physical activity/exercise could be considered for maintaining cognition. In people with moderate dementia, exercise could be considered for stabilizing disability compared to usual care.

*Quality of evidence*: Exercise: very low ⊕ for cognitive outcomes; low ⊕  ⊕ for disability.

*Strength of recommendation*: Strong for intervention ↑↑.

##### Additional information/secondary outcomes

Some data were available for secondary outcomes, relevant from a clinical perspective. Overall, physical activity/exercise improved depressive symptoms in moderate dementia (mean MMSE = 17.5, range 7.3–23.8) (SMD = – 0.18; 95% CI – 0.33 to – 0.02; moderate certainty of evidence) and BPSD (mean MMSE = 17.6, range 9.7–23.8) (MD = – 4.62; 95% CI – 9.08 to – 0.16; very low certainty of evidence). Of importance, in people with moderate dementia (mean MMSE = 19.8), physical activity/exercise interventions significantly decreased the risk (RR = 0.69; 95% CI 0.55–0.86) and the number of falls (MD = – 1.06; 95% CI – 1.67 to – 0.46), with a certainty of evidence low to moderate. On the contrary, physical activity/exercise did not decrease the risk of hospitalization, mortality and did not improve quality of life.

Regarding findings present in systematic reviews without a formal meta-analysis with low risk of bias according to the ROBIS, aerobic exercise improved only some cognitive outcomes [52], while a mixed physical activity/exercise intervention improved executive function in four RCTs, in people with AD (low risk of bias in the ROBIS evaluation) [53]. Three systematic reviews [54–56] (two high risk of bias and one low risk according to the ROBIS) reported that mixed and home-based physical activity improved several cognitive (global and specific) and non-cognitive (such as BPSD, quality of life, disability, and physical function tests) outcomes in people with dementia.

##### Expert consensus statement

86% of the experts agreed that physical activity/exercise is of importance for maintaining cognitive reserve and function in people with dementia (Table 6). In people living with dementia, physical activity/exercise may have beneficial effects on non-cognitive neuropsychiatric symptoms, such as mood, but these potential benefits should be balanced compared to potential side effects (Table 7).

**Table 6:** Effect of exercise on cognitive outcomes and disability in dementia

Certainty assessment							No. of patients		Effect		Certainty	Importance
No. of studies	Study design	Risk of bias	Inconsistency	Indirectness	Imprecision	Other considerations	Intervention	Standard care	Relative (95% CI)	Absolute (95% CI)		
Global cognition (in AD) (mixed physical activity intervention)												
13	Randomized controlled trials	Very serious^a^	Very serious^b^	Not serious	Not serious	Publication bias strongly suspected^c^	342	331	–	SMD 1.1 SD higher (0.65 higher to 1.64 higher)	⨁◯◯◯ Very low	Critical
Global cognition (in dementia) (mixed physical activity intervention)												
19	Randomized controlled trials	Not serious	Very serious^b^	Not serious	Not serious	None	433	405	–	SMD 0.48 SD higher (0.22 higher to 0.74 higher)	⨁⨁◯◯ Low	Critical
ADL (physical activity interventions home-based)												
3	Randomized controlled trials	Not serious	Serious^a^	Not serious	Serious^b^	None	94	86	–	SMD 0.77 SD higher (0.17 higher to 1.37 higher)	⨁⨁◯◯ Low	Critical
Disability in ADL (in dementia) (physical activity mixed interventions)												
11	Randomized controlled trials	Very serious^d^	Very serious^e^	Not serious	Not serious	None	730	581	–	SMD 0.5 SD higher (0.03 lower to 1.02 higher)	⨁◯◯◯ Very low	Critical

*CI* Confidence interval, *AD* Alzheimer’s disease, *SMD* Standardized mean difference, *SD* Standard deviation, *ADL* Activities of daily living

^a^One or more of the three criteria (randomization, masking, drop-out rate > 30%) is not met in > 30% of trials included

^b^*I*^2^ >  = 75%

^c^Egger’s test (*p* value) < 0.0001

^d^One or more of the three criteria (randomization, masking, drop-out rate < 30%) is not met in 10–30% of trials included

^e^*I*^2^ between 50% and 75%

**Table 7:** Effect of exercise on secondary outcomes in dementia

Certainty assessment							No. of patients		Effect		Certainty	Importance
No. of studies	Study design	Risk of bias	Inconsistency	Indirectness	Imprecision	Other considerations	Physical activity/exercise	Standard care	Relative (95% CI)	Absolute (95% CI)		
Depressive symptoms in dementia (physical activity mixed interventions)												
15	Randomized controlled trials	Not serious	Not serious	Not serious	Not serious	Publication bias strongly suspected^c^	707	722	–	SMD 0.18 SD lower (0.33 lower to 0.02 lower)	⨁⨁⨁◯ Moderate	Important
Behavioral and psychological symptoms in dementia BPSD (physical activity mixed interventions)												
6	Randomized controlled trials	Very serious^d^	Very serious^e^	Not serious	Not serious	None	497	564	–	MD 4.62 SD lower (9.08 lower to 0.16 lower)	⨁◯◯◯ Very low	Important
Risk of falls in dementia (physical activity interventions home-based)												
2	Randomized controlled trials	Not serious	Not serious	Not serious	Serious^b^	Publication bias strongly suspected^c^	Not available	Not availaible	RR 0.69 (0.55–0.86)	Not possible	⨁⨁◯◯ Low	Important
Number of falls in dementia (physical activity interventions home-based)												
3	Randomized controlled trials	Not serious	Not serious	Not serious	Serious^b^	None	137	137	–	MD 1.06 lower (1.67 lower to 0.46 lower)	⨁⨁⨁◯ Moderate	Important
Risk of falls in dementia (physical activity mixed interventions)												
3	Randomized controlled trials	Not serious	Not serious	Not serious	Serious^b^	None	60/134 (44.8%)	90/137 (65.7%)	RR 0.69 (0.55–0.85)	204 fewer per 1.000 (from 296 to 99 fewer)	⨁⨁⨁◯ Moderate	Important
Depressive symptoms (in AD) (physical activity mixed interventions)												
3	Randomized controlled trials	Very serious^d^	Not serious	not serious	serious^b^	None	110	109	–	SMD 0.18 SD higher (0.03 lower to 0.39 higher)	⨁◯◯◯ Very low	Important
Mortality (in dementia) (physical activity mixed interventions)												
10	Randomized controlled trials	Very serious^d^	Not serious	Not serious	Not serious	None	25/341 (7.3%)	27/348 (7.8%)	RR 0.66 (0.43–1.02)	26 fewer per 1.000 (from 44 fewer to 2 more)	⨁⨁◯◯ Low	Important
Length of stay in hospital (in dementia) (physical activity mixed interventions)												
3	Randomized controlled trials	Very serious^d^	Not serious	Not serious	Not serious	None	207	205	–	MD 0.16 lower (0.36 lower to 0.03 higher)	⨁⨁◯◯ Low	Important
BPSD (in dementia) (physical activity mixed interventions)												
3	Randomized controlled trials	Very serious^d^	Serious^a^	Not serious	Serious^b^	None	145	136	–	MD 3.89 lower (8.97 lower to 1.2 higher)	⨁◯◯◯ Very low	Important
Apathy (in dementia) (physical activity mixed interventions)												
3	Randomized controlled trials	Serious^f^	Serious^a^	Not serious	Serious^b^	None	117	111	–	SMD 0.34 SD lower (0.83 lower to 0.15 higher)	⨁◯◯◯ Very low	Important
Anxiety (in dementia) (physical activity mixed interventions)												
3	Randomized controlled trials	Serious^d^	Serious^a^	Not serious	Serious^b^	Publication bias strongly suspected^c^	109	101	–	SMD 0.33 SD lower (0.84 lower to 0.18 higher)	⨁◯◯◯ Very low	Important
Quality of life (in dementia) (physical activity mixed interventions)												
6	Randomized controlled trials	Serious^f^	Very serious^e^	Not serious	Not serious	None	385	380	–	SMD 0.33 SD higher (0.2 lower to 0.86 higher)	⨁◯◯◯ Very low	Important
Rate of Hospitalization (in dementia) (physical activity mixed interventions)												
5	Randomized controlled trials	Very serious^d^	Not serious	Not serious	Not serious	None	101/299 (33.8%)	95/294 (32.3%)	RR 1.05 (0.85–1.31)	16 more per 1.000 (from 48 fewer to 100 more)	⨁⨁◯◯ Low	Important

*CI* confidence interval, *MD* mean difference, *RR* risk ratio, *SMD* Standardized mean difference, *SD* Standard deviation, *AD* Alzheimer’s disease, *BPSD* Behavioral And Psychological Symptoms In Dementia

^a^*I*^2^ between 50% and 75%

^b^Sample size less than 400 participants

^c^Egger's test (*p* value) < 0.05

^d^One or more of the three criteria (randomization, masking, drop-out rate > 30%) is not met in > 30% of trials included

^e^*I*^2^ more than 75%

^f^One or more of the three criteria (randomization, masking, drop-out rate > 30%) is not met in 10–30% of trials included

##### Future research directions

- In people with dementia who have traditionally been excluded from trials, such as those with severe forms of dementia, studies exploring the effect of physical activity and exercise are encouraged (Table 8).
- Studies including less common forms of dementia, such as frontotemporal and Lewy Body dementia, are needed.

**Table 8:** Effect of exercise on secondary outcomes in dementia: narrative findings

Intervention	Population	Outcome	Number of studies	Main findings
Aerobic exercise	AD	Cognitive function	8	There is scarce evidence that aerobic exercise improves cognition in AD patients. Overall, the included studies reported only positive effects for patients’ global cognition after intervention, mainly due to a lack of accurate neuropsychological assessment of each cognitive domain
PA mixed	AD	Executive function	4	Significant improvement was seen in all studies
PA home-based	Dementia	BPSD	7	Small effect on BPSD (ES = − 0.37, 95% CI − 0.57, − 0.017)
PA home-based	Dementia	Carer's burden	3	Medium reduction on carer's burden (ES = − 0.63, 95% CI − 0.94, − 0.32) for NPI Caregivers subscale and low and negative (ES = − 0.45, 95% CI − 0.77, − 0.13) for ZBI
PA home-based	Dementia	Cognitive function	6	Medium effect on MMSE (ES = 0.71, 95% CI 0.43, 0.99)
PA home-based	Dementia	Disability	4	Important effect on disability (ES = 0.80, 95% CI 0.53, 1.07)
PA home-based	Dementia	Health-related physical fitness	6	Large effect on physical tests Functional Reach test (ES = 2.24, 95% CI 1.80, 2.68), TUG test (ES = − 2.40, 95% CI − 2.84, − 1.96)
PA home-based	Dementia	QoL	2	Small effect on QoL
PA mixed	Dementia	Physical performance test	10	Lower-limb strength improved equally in multicomponent interventions and progressive resistance training
PA mixed	Dementia	BPSD	3	All three RCTs reported significant reductions of BPSD and differences in comparison with the pre-test and control groups
PA home-based	Dementia home	Disability	7	Significant effect of physical activity on functional ability, particularly on mobility items
PA home-based	Dementia home	Mobility	7	Significant effect of physical activity on functional ability, particularly on mobility items
PA mixed	Dementia nursing home	Cognitive function	7	Among 7 RCTs initially included, physical activity improved cognitive measures in two
PA mixed	Dementia nursing home	Mood and Depression	5	Not clear effect on depression and mood measures
PA mixed	Dementia nursing home	Functional ability	5	Significant effect of physical activity on functional ability, particularly on mobility items
PA mixed	Dementia nursing home	Mobility	5	Significant effect of physical activity on functional ability, particularly on mobility items
PA mixed	Dementia nursing home	Cognitive function	5	There is moderate-to-strong evidence that physical activity can effectively maintain cognitive function in nursing home residents with Dementia
PA mixed	Moderate severe dementia	Disability	5	In one high-quality study over five, physical activity programs significantly delayed deterioration of ADL performance

*PA* physical activity, *AD* Alzheimer’s disease, *BPSD* Behavioral and Psychological Symptoms in Dementia, *ES* Effect Size, *CI* Confidence interval, *NPI* Neuropsychiatric Inventory, *ZBI* Zarit Burden Interview, *MMSE* Mini-Mental State Examination, *TUG* Timed Up and Go, *QoL* Quality of Life, *RCTs* Randomized Controlled Trials, *ADL* Activities of daily living

## Discussion

**Fig. 1:**
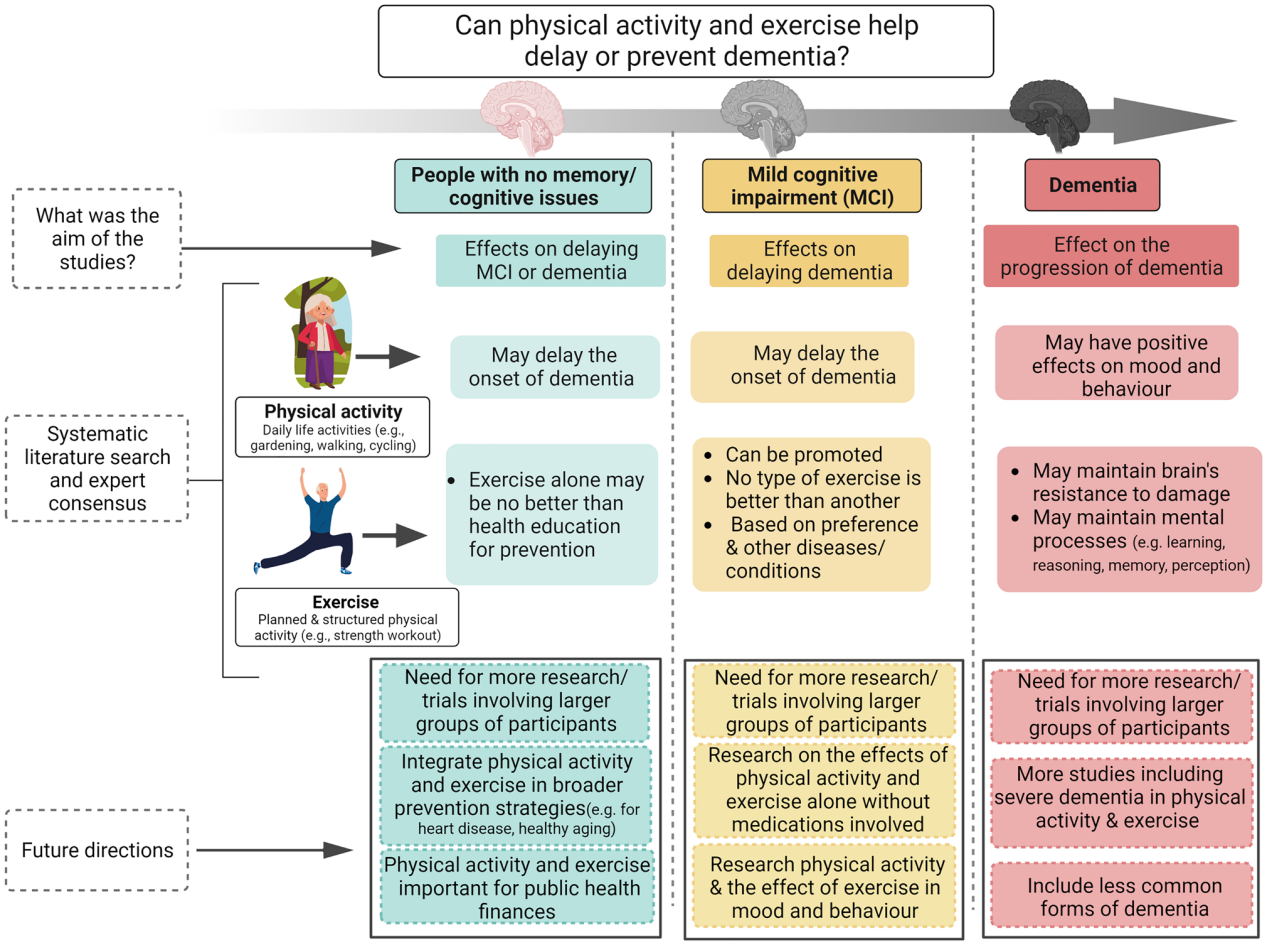
Infographic regarding the effect of physical activity and exercise in people without cognitive impairment, in mild cognitive impairment and in dementia. Created with Biorender.com and vecteezy.com

In these guidelines, derived from the literature review and reported using GRADE framework and the discussion of the societies’ experts to reach a consensus, we summarized the evidence of the effect of physical activity and exercise for the prevention and management of MCI and dementia, as summarized in the infographic (Fig. 1). Overall, our guidelines strongly recommend the use of physical activity and exercise for the prevention and management of MCI and dementia, although the evidence is not conclusive, and it has a low to very low quality.

### Pathophysiological mechanisms supporting the benefits of physical activity and exercise in cognition

The finding of some positive effects of physical activity and exercise in the prevention and management of dementia can be justified by several hypotheses. First, higher physical activity and exercise levels are associated with a lower incidence of common risk factors for dementia, such as cardiovascular diseases [57, 58]. This effect is probably mediated by the modulation of some neurotrophic factors (e.g., brain-derived neurotrophic factor that that may promote neuronal survival in some brain regions, such as the hippocampus) [59] and by the decrease in inflammation [60] and insulin resistance [61]. Moreover, physical activity and exercise are likely associated with positive structural and functional brain changes, as shown by some studies using functional Magnetic Resonance Imaging, particularly in those regions more involved in cognition, such as the hippocampus, for which the effects of exercise on hippocampal volume were modest, but statistically significant [62]. Even if the main focus of the human volumetry work has largely been on the hippocampus, higher exercise and physical activity probably could mediate the activity of other brain regions, such as the prefrontal cortex and cortical thickness, involved in cognition [63]. Finally, physical activity and exercise can positively affect some behavioral/socioemotional aspects such as sleep, depression and anxiety, strongly associated with dementia and MCI [57].

The experts of the board also raised the important issue of the different effects of physical activity and exercise on the outcomes considered in these guidelines. The potential explanation of this finding is probably that physical activity is an umbrella term that refers to movement that increases energy expenditure independently of its intent or intensity, measured in observational studies included in this work as self-reported information (e.g., questionnaires), while exercise is usually implemented in the framework of RCTs, which are fewer and usually include samples that are much smaller compared to those included in observational studies [64]. Physical activity seems to have a positive effect that often is not confirmed for exercise likely introducing a methodological point, i.e., physical activity has been mainly studied in observational studies, which are more prone to bias, while exercise has been evaluated in RCTs that, however, probably did not have the power or duration to investigate its relationship with MCI/dementia, particularly when talking about prevention.

Finally, as also declared in recommendations given by the experts, MCI and dementia are typical multifactorial conditions. Therefore, these conditions are probably related to multiple risk factors, such as smoking, obesity, low formal education level, hearing loss and many others [4, 65]. In this respect, physical activity and exercise should be implemented together with other interventions [66], e.g. correct nutrition [67], smoking cessation, and others [68, 69].

### Facilitators and barriers to application

We believe that the distribution and implementation of our guidelines, based on one of the most important non-pharmacological approaches, i.e., physical activity/exercise, could have several facilitators. Among them, probably the most important is Alzheimer Europe, an umbrella organization of 41 national Alzheimer's associations coming from 37 European countries. (https://www.alzheimer-europe.org/), as well as the range of other partner societies, including dementia, geriatrics and other specialists. We will distribute these guidelines, send them to the representatives of all national societies that belong to the European societies to cover medical and non-medical specialists in dementia and facilitate the effective implementation of the guidelines. The guidelines will be translated into national languages to facilitate wider dissemination. Second, we will include an infographic and other graphical supports that can effectively inform individuals interested in dementia while avoiding scientific jargon that may be unfamiliar to non-experts. Moreover, a plain language summary revised by lay representatives has been developed. Third, a pilot test in Italy will be performed to check potential issues with national societies of the disciplines involved and with lay representatives. Fourth, we will prepare several in-person and online meetings during congresses (international and national) to inform professionals and stakeholders. Finally, we plan to update this work in 5 years, including new questions and updating the evidence.

### Monitoring/auditing criteria

We will monitor the guidelines implementation with regular feedback (once a year) regarding this project by contacting steering group members of the national people responsible for this project across Europe.

### Limitations

Our ambitious attempt at creating clinical practice guidelines for physical activity and exercise for MCI and dementia based on an International consensus of experts, mainly European, and other stakeholders is not free of limitations. Although we believe that the team of experts involved covers many of the relevant disciplines regarding cognitive issues and physical activity/exercise, some disciplines are missing, such as general practitioners or psychologists. Second, the input from older adults with dementia/MCI derives from lay representatives and not from people living with these conditions themselves. Although we aimed for our recommendations to be pragmatic and simple to apply and adaptable to older persons’ needs, no formal testing and validation was performed. Third, further research in this area is not only important for overcoming some weaknesses that we found in our analyses, but also for exploring the characteristics of physical activity/exercise that are more important to achieve the potential benefits on cognitive status [70]. Similarly, it would be of interest to investigate if some methods, such as self-management of physical activity and exercise [71], can better highlight the importance of physical activity/exercise in this area. Finally, a consistent part of the works included was supported by a low methodological quality.

### Unanswered questions

These guidelines indicate that several questions remain unanswered. First, although our protocol aimed to extract comprehensive information, we often encountered poorly described and/or heterogenous relevant details regarding physical activity/exercise, such as type, frequency or intensity, and therefore, this could limit the practical diffusion of our work. It should be acknowledged that there is significant individual variation in the uptake of physical activity/exercise, and that these are often lifelong behaviors, and that encouraging and maintaining these as new behaviors requires learning from experts in the different fields (physical activity, psychology, behavior change), as well as ‘experts by experience’, and particularly when considering people affected by MCI or dementia. Second, particularly for dementia, we were not able to indicate the effectiveness of physical activity/exercise graded according to severity of dementia or by pathological subgroups of dementia. In this sense, less common forms of dementia or milder as well as more severe stages of dementia are practically uncovered by our guidelines indicating the need for future studies specifically tailored for these patients. Third, the potential for prevention is high and might be higher in low-income and middle-income countries (LMIC), where the majority of dementia cases will occur in the next years. Finally, despite being planned, we were unable to extract any data regarding quality of life as it was not included as an outcome in MCI or dementia studies.

## Concluding remarks

Our consensus agreed to support physical activity and exercise in our guidelines after taking into account their overall beneficial effect on our target population's global health, including physical and psychological health. Therefore, even in the presence of a faint evidence base for positive cognitive effects physical activity and exercise, we believe that they should be recommended. We hope that our guidelines will help not only the physicians, but also all the people taking care of people affected by cognitive disorders, including the caregivers.

### Supplementary Information

Below is the link to the electronic supplementary material.

Supplementary file1 (DOCX 177 KB)

## Data Availability

Databases are available upon reasonable request to the corresponding author.
