# Supplementary material for: Physical activity and exercise for the prevention and management of mild cognitive impairment and dementia: a collaborative international guideline
Source: Eur Geriatr Med. 2023 Sep 28;14(5):925–52. doi: 10.1007/s41999-023-00858-y (PMC10587099; doi:10.1007/s41999-023-00858-y)

**Supplementary Table 1. Participants in the guidelines**

| **SURNAME** | **FIRST NAME** | **SOCIETY** |
| --- | --- | --- |
| Agar | Brugiavini | European Interdisciplinary Council on Ageing |
| Alonso Ramirez | Javier | European Geriatric Medicine Society |
| Alves | Mariana | European Geriatric Medicine Society |
| Bahat | Gülistan | European Geriatric Medicine Society |
| Barbagallo | Mario | International Association of Gerontology and Geriatrics-European Region |
| Bauer | Jurgen | European Geriatric Medicine Society |
| Bautman | Ivan | European Geriatric Medicine Society |
| Bruyere | Olivier | European Society of Clinical and Economic Aspects of Osteoporosis and Osteoarthritis |
| Buzaco | Rui | European Geriatric Medicine Society |
| Casas Herrero | Álvaro | European Geriatric Medicine Society |
| Cesari | Matteo | European Geriatric Medicine Society |
| CHEN | Yaohua | European Geriatric Medicine Society |
| Cherubini | Antonio | European Geriatric Medicine Society |
| Christodoulou | Nikos | World Psychiatry Association-Preventive Psychiatry section |
| Corbi | Graziamaria | European Geriatric Medicine Society |
| Cruz Jentoft | Alfonso | European Geriatric Medicine Society |
| De Cock | Anne-Marie | European Geriatric Medicine Society |
| Demurtas | Jacopo | European Geriatric Medicine Society |
| DOGU | Burcu Balam | European Geriatric Medicine Society |
| Duraes | Joao | European Academy of Neurology |
| Freiberger | Ellen | European Geriatric Medicine Society |
| Fusar-Poli | Paolo | European College of Neuropsychopharmacology |
| Georges | Jean | Alzheimer Europe |
| Haaksma | Miriam L. | European Geriatric Medicine Society |
| Karpenko | Olga | World Psychiatry Association-Preventive Psychiatry section |
| Kotsani | Marina | European Geriatric Medicine Society |
| Lamb | Sarah | European Geriatric Medicine Society |
| Lamloum | Mounir | European Geriatric Medicine Society |
| Lappas | Andreas | World Psychiatry Association-Preventive Psychiatry section |
| Limongi | Federica | European Interdisciplinary Council on Ageing |
| Liuu | Evelyne | European Geriatric Medicine Society |
| Maggi | Stefania | European Interdisciplinary Council on Ageing |
| Melis | Rene | European Geriatric Medicine Society |
| Pinto | Daniel | European Society of Clinical and Economic Aspects of Osteoporosis and Osteoarthritis |
| Perez Bazan | Laura Monica | European Geriatric Medicine Society |
| Polidori | Maria Cristina | European Geriatric Medicine Society |
| Quinn | Terence J | Cochrane Dementia and Cognitive Improvement: |
| Ramalho | Rodrigo | World Psychiatry Association-Preventive Psychiatry section |
| Reginster | Jean-Yves | European Society of Clinical and Economic Aspects of Osteoporosis and Osteoarthritis |
| Ricart | Joan Ars | European Geriatric Medicine Society |
| Rincon | Almudena Medina | European Geriatric Medicine Society |
| Rolland | Yves | European Geriatric Medicine Society |
| Romero-Ortuno | Roman | European Geriatric Medicine Society |
| Sacco | Guillaume | European Geriatric Medicine Society |
| Schlögl | Mathias | European Geriatric Medicine Society |
| Schmidt | Reinhold | European Academy of Neurology |
| Schoene | Daniel | European Geriatric Medicine Society |
| Shapiro | Debbie | European Geriatric Medicine Society |
| Shenkin | Susan | European Geriatric Medicine Society |
| Sieber | Cornel | European Geriatric Medicine Society |
| Smith | Lee | European Geriatric Medicine Society |
| Solmi | Marco | European College of Neuropsychopharmacology |
| Soysal | Pinar | European Geriatric Medicine Society |
| Steen Frederiksen | Kristian | European Academy of Neurology |
| TANNOU | Thomas | European Geriatric Medicine Society |
| Veronese | Nicola | European Geriatric Medicine Society |
| YILMAZ | Ozlem | European Geriatric Medicine Society |

**Supplementary Table 2. Topic 1: Prevention**

| **Component** | **Description** |
| --- | --- |
| **Review questions** | In people without dementia or mild cognitive impairment, are physical activity and/or exercise able to delay the onset of dementia and/or mild cognitive impairment? |
| **Objective** | To investigate the effect and the safety of physical activity/exercise in the prevention of dementia and MCI |
| **Population** | People without dementia/MCI, defined according to validated criteria, validated scales, (electronic) health records. |
| **Interventions** | Physical exercise regimens (in intervention studies); higher physical activity level (in observational studies) as the highest quantile available |
| **Comparisons** | Inactive (usual care, standard care, watching list) (in intervention studies); lower physical activity level (in observational studies) as the lowest quantile available |
| **Primary outcomes** | Incidence of dementia (defined according to validated criteria [National Institute of Neurological and Communicative Disorders and Stroke and the Alzheimer's Disease and Related Disorders Association criteria, Diagnostic and Statistical Manual of mental disorders, International Classification of Diseases], validated scales, (electronic) health records) |
| **Secondary outcomes** | - MCI and MCI/dementia - Adverse events (total and specific) and safety measures - Drop-out rate - Disability in ADL (activities of daily living)/IADL (instrumental activities of daily living) - Cognitive measures: global and specific domains of cognition, i.e., attention, executive function, memory, motor speed, and language); - Quality of life |
| **Study design** | Hierarchical: systematic reviews (with or without meta-analyses) that synthesize randomized controlled trials (RCTs) or controlled clinical trials (CCTs) will be the priority. If not available, singular RCTs/CCTs will be preferred to observational studies ( following the order: cohort, nested case-control). If a systematic review-meta-analysis was done before two years from the search, we will update the search with the novel data. |
| **Exclusion criteria** | Diagnosis of dementia or MCI made with non-standardized criteria  Conference abstracts  Cross sectional studies |
| **Planned subgroups analyses** | Study design (cohort vs. nested case-control studies)  Intervention (RCTs) vs. observational studies |

**Topic 2: Mild cognitive impairment**

| **Component** | **Description** |
| --- | --- |
| **Review questions** | Are physical activity and exercise able to delay the onset of dementia in people with mild cognitive impairment? |
| **Objective** | To verify the effect and the safety of physical activity/exercise in the prevention of dementia in people with MCI |
| **Population** | Mild cognitive impairment, defined according to validated criteria [Diagnostic and Statistical Manual of mental disorders, International Classification of Diseases], validated scales, (electronic) health records. |
| **Interventions** | Physical exercise regimens (in intervention studies); high physical activity level (in observational studies) |
| **Comparisons** | Inactive (usual care, standard care, watching list) (in intervention studies); lower physical activity level (in observational studies) |
| **Primary outcomes** | Rate of conversion in dementia (defined according to validated criteria, validated scales, (electronic) health records) |
| **Secondary outcomes** | - Adverse events (total and specific) and safety measures - Drop-out rate - Disability in ADL/IADL - Cognitive measures: global and specific domains of cognition, i.e., attention, executive function, memory, motor speed, and language); - Quality of life |
| **Study design** | Hierarchical: systematic reviews (with or without meta-analyses) that synthesize randomized controlled trials (RCTs) or controlled clinical trials (CCTs) will be the priority. If not available, singular RCTs/CCTs will be preferred to observational studies (following the order: cohort, nested case-control). If a systematic review-meta-analysis was done before two years from the search, we will update the search with the novel data. |
| **Exclusion criteria** | People with dementia.  Active controls (e.g., nutritional interventions) |
| **Planned subgroups analyses** | Type of physical exercise intervention  Type of physical activity  Type of MCI (anamnestic, non-anamnestic, others)  Study design |

**Topic 3: Dementia**

| **Component** | **Description** |
| --- | --- |
| **Review questions** | Is physical exercise able to improve cognition and disability in people with dementia? |
| **Objective** | To verify the effect and the safety of physical exercise in improving cognition and disability in people with dementia |
| **Population** | People with dementia, defined according to validated criteria (defined according to validated criteria [National Institute of Neurological and Communicative Disorders and Stroke and the Alzheimer's Disease and Related Disorders Association criteria, Diagnostic and Statistical Manual of mental disorders, International Classification of Diseases]), validated scales, (electronic) health records. |
| **Interventions** | Physical exercise regimens |
| **Comparisons** | Inactive (e.g., usual care, standard care, watching list) |
| **Primary outcomes** | Cognitive tests (global and specific domains of cognition, i.e., attention, executive function, memory, motor speed, and language); disability in ADL/IADL |
| **Secondary outcomes** | - Adverse events (total and specific) - Drop-out rate - Mortality - Quality of life - Physical performance tests (e.g., gait speed, chair stands time, balance, short physical performance battery, handgrip strength) - Caregivers’ burden (Caregiver Burden Inventory) - BPSD - Sleep quality (Pittsburgh Sleep Quality Index) |
| **Study design** | Hierarchical: systematic reviews (with or without meta-analyses) that synthesize randomized controlled trials (RCTs) or controlled clinical trials (CCTs) will be the priority. If not available, singular RCTs/CCTs will be used. If a systematic review-meta-analysis was done before two years from the search, we will update the search with the novel data. |
| **Planned subgroups analyses** | Type of physical exercise intervention  Type of physical activity  Type of dementia (any, Alzheimer, vascular dementia, Lewy, fronto-temporal, etc.)  Study design |
| **Exclusion criteria** | Conference abstracts  Active controls (e.g., nutritional interventions)  No standardized criteria for dementia |

**Supplementary Table 3. Criteria evidence for the GRADE of the randomized controlled trials included.**

| **Downgrade** | **Risk of bias** | **Inconsistency** | **Indirectness** | **Imprecision** | **Publication bias** |
| --- | --- | --- | --- | --- | --- |
| **-1** | If one or more of the three criteria (randomization, masking, drop-out rate <30%) is not met in **10-30%** of trials included in the systematic review | **I^2^ 50-74%** | The question being addressed by the guideline panel is **different** from the available evidence regarding the PICO or regarding the characteristics of those who will deliver the intervention | (a)The **overall number** of individuals included in trials is low (less than **400 individuals,** both treatment arms) **OR** (b) the 95% confidence interval includes both 1) no effect and 2) appreciable benefit (RR: ≤0.75) or appreciable harm (RR: ≥1.25) ^a^ | - |
| **-2** | If one or more of the three criteria (randomization, masking, drop-out rate>30%) is not met in **>30%** of trials included in the systematic review | **I^2^ > 75%** | The question being addressed by the guideline panel is **markedly different** from the available evidence regarding the PICO or regarding the characteristics of those who will deliver the intervention | (a) the **overall number** of individuals included in trials is **very low** (**less than 200 individuals,** both treatment arms) **AND** (b) the 95% confidence interval includes both 1) no effect and 2) appreciable benefit (RR: ≤0.75) or appreciable harm (RR: ≥1.25) ^a^ | **Egger’s test** (p-value) <0.05 |

^a^ For **continuous outcomes** “***no effect***” means a SMD with a confidence interval that ***crosses zero***; **appreciable** benefit or appreciable harm means that the **upper or lower confidence limit crosses an effect size of 0.5** in either direction.

For **dichotomous outcomes** “***no effect***” means an estimate with a confidence interval that ***crosses one***; **appreciable** benefit or appreciable harm means that the upper or lower confidence limit **crosses a risk of 1.25 or 0.75**.

**Abbreviations: OR:** Odds ratio**; PICO:** Population, Intervention, Comparison and Outcomes; **RR**: Risk Ratio

**Supplementary Table 4. Search strategy**

| 1. exp Exercise/  2. Exercise Therapy/  3. Dancing/  4. Dance Therapy/  5. Occupational Therapy/  6. Physical Conditioning, human/  7. Physical Fitness/  8. Exercise Movement Techniques/  9. Physical Endurance/  10. Physical Exertion/  11. Physical Therapy/  12. Resistance Training/  13. Tai Ji/  14. aerobic*.ti,ab.  15. (dance or dancing).ti,ab.  16. ((exercis* adj3 train*) or exercising).ti,ab.  17. (exercis* adj3 prog*).ti,ab.  18. "exercise bike*".ti,ab.  19. (exercis* adj3 cardiovascular).ti,ab.  20. exergam*.ti,ab.  21. "exercise gaming".ti,ab.  22. "exer-gam*".ti,ab.  23. jogging.ti,ab.  24. occupational therapy.ti,ab.  25. "physical activit*".ti,ab.  26. "physical exercis*".ti,ab.  27. "physical endurance".ti,ab.  28. "physical performance".ti,ab.  29. physiotherapy.ti,ab.  30. (resistance adj2 train*).ti,ab.  31. (stretch adj4 (muscle* or exercis*)).ti,ab.  32. swim*.ti,ab.  33. treadmill.ti,ab.  34. walking.ti,ab.  35. yoga.ti,ab.  36. exp Dementia/  37. *Cognition/  38. Cognitive Dysfunction/  39. *Cognition Disorders/  40. Memory Disorders/  41. dement*.ti,ab.  42. alzheimer*.ti,ab.  43. "mental perform*".ti,ab.  44. "preclinical AD".ti,ab.  45. "pre-clinical AD".ti,ab.  46. ((cognit* or memory or cerebr* or mental*) adj3 (declin* or impair* or los* or deteriorat* or degenerat* or complain* or disturb* or disorder* or insufficient* or chronic)).ti,ab.  47. MCI.ti,ab.  48. aMCI.ti,ab.  49. MCIa.ti,ab.  50. (lewy* adj2 bod*).ti,ab.  51. nondemented.ti,ab.  52. non-demented.ti,ab.  53. (cognitively healthy or cognitively normal).ti,ab.  54. Posterior cortical atrophy.ti,ab.  55. binswanger*.ti,ab.  56. Progressive supranuclear palsy.ti,ab.  57. Steele-Richardson-Olszewski syndrome.ti,ab.  58. Huntington*.ti,ab.  59. Frontotemporal disorder*.ti,ab.  60. Frontotemporal degeneration.ti,ab.  61. Corticobasal degeneration.ti,ab.  62. Corticobasal syndrome.ti,ab.  63. or/1-35  64. or/36-62  65. 63 and 64  66. Meta-Analysis as Topic/  67. meta analy*.tw.  68. metaanaly*.tw.  69. Meta-Analysis/  70. (systematic adj (review$1 or overview$1)).tw.  71. exp Review Literature as Topic/  72. or/66-71  73. cochrane.ab.  74. embase.ab.  75. (psychlit or psyclit).ab.  76. (psychinfo or psycinfo).ab.  77. (cinahl or cinhal).ab.  78. science citation index.ab.  79. bids.ab.  80. cancerlit.ab.  81. or/73-80  82. reference list$.ab.  83. bibliograph$.ab.  84. hand-search$.ab.  85. relevant journals.ab.  86. manual search$.ab.  87. or/82-86  88. selection criteria.ab.  89. data extraction.ab.  90. 88 or 89  91. Review/  92. 91 and 90  93. 72 or 81 or 87 or 92  94. 65 and 93  95. randomized controlled trial.pt.  96. controlled clinical trial.pt.  97. randomized.ab.  98. placebo.ab.  99. randomly.ab.  100. trial.ab.  101. groups.ab.  102. or/95-101  103. 65 and 102  104. Epidemiologic studies/  105. exp case control studies/  106. exp cohort studies/  107. Case control.tw.  108. (cohort adj (study or studies)).tw.  109. Cohort analy$.tw.  110. (Follow up adj (study or studies)).tw.  111. (observational adj (study or studies)).tw.  112. Longitudinal.tw.  113. Retrospective.tw.  114. Cross sectional.tw.  115. Cross-sectional studies/  116. or/104-115  117. 65 and 116  118. 94 or 103 or 117 |
| --- |

**Supplementary Table 5. Plan language summary for patients and caregivers**

It is estimated that every three seconds one person in the world will be with dementia. Currently, dementia is not treatable with pharmacological approaches. Therefore, an increasing interest is present for potential modifiable risk factors for this condition, such as physical inactivity. With these guidelines, we try to give some practical indications for the prevention and the treatment of dementia, also in early stages such as mild cognitive impairment (MCI), using physical activity and exercise as intervention.

Overall, our guidelines support the importance of higher physical activity for delaying the onset of dementia in people initially free from this condition. Moreover, in patients already with MCI, exercise may be used for maintaining cognition. Finally, in people already living with dementia, exercise may be used for managing cognition and disability. However, we need more studies for better supporting these statements.

Physical activity/exercise should be recommended also in view of their multiple beneficial effects outside cognition, as recognized by the experts involved in these guidelines. Our guidelines suggest that physical activity/exercise may improve cognitive aspects in dementia and MCI and could be used for the prevention of these conditions often associated with poor quality of life and disability, even if the experts suggest that the integration with pharmacological and non-pharmacological approaches can further increase the efficacy of physical activity/exercise.

**Supplementary Figure 1. ROBIS quality assessment of the included systematic reviews**


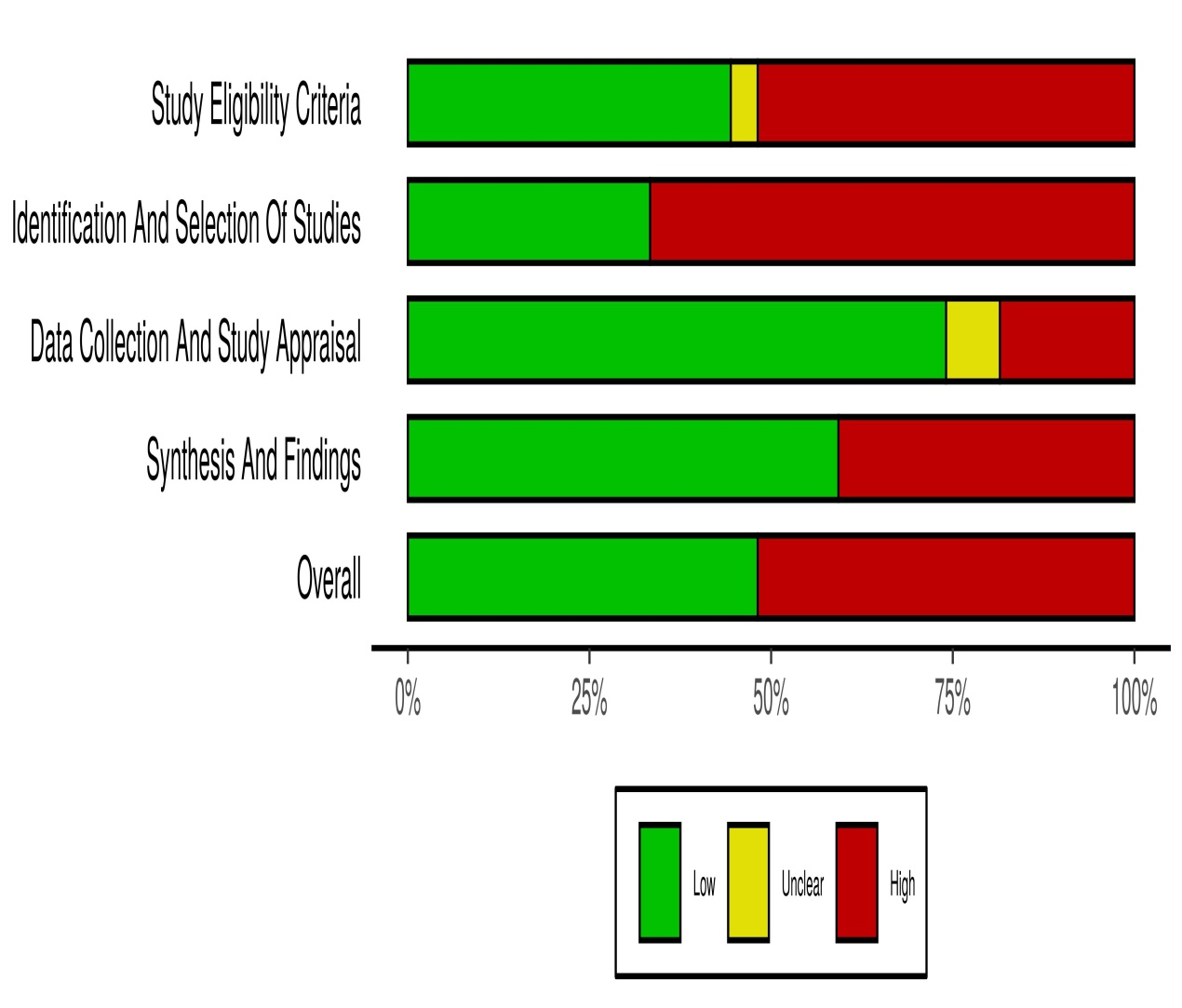

Supplement: Supplementary file 1 — Supplementary file1 (DOCX 177 KB) [file 41999_2023_858_MOESM1_ESM.docx]
